# CEST MRI Processing Pipeline in Pilot Study of Alzheimer's Disease Patients

**DOI:** 10.1002/mrm.70240

**Published:** 2026-01-04

**Authors:** Alexander Asturias, Fang Frank Yu, Elizabeth M. Davenport, Brendan J. Kelley, Ivan E. Dimitrov, Jochen Keupp, Elena Vinogradov

**Affiliations:** ^1^ Department of Radiology University of Texas Southwestern Medical Center Dallas Texas USA; ^2^ Advanced Imaging Research Center University of Texas Southwestern Medical Center Dallas Texas USA; ^3^ Department of Neurology University of Texas Southwestern Medical Center Dallas Texas USA; ^4^ Philips Cambridge Massachusetts USA; ^5^ Philips Innovative Technologies Hamburg Germany

## Abstract

**Purpose:**

To develop a processing pipeline combining neuroimaging analysis tools with CEST postprocessing and utilize it in a pilot study probing differences between cognitively impaired (CI) Alzheimer's disease (AD) patients and cognitively normal (CN) individuals.

**Methods:**

Eight subjects (4 biomarker confirmed CI with underlying AD and 4 CN) were scanned on a 3T MRI scanner. The processing pipeline included rigid motion correction and co‐registration of the CEST images with the 3D T_1w_ images using Advanced Normalization Tools (ANTs). MTR_asym_ maps at 1 ppm, 2 ppm and 3.5 ppm were generated on a voxel‐by‐voxel basis. Each subject's 3D T1w images were processed with FreeSurfer to generate brain region specific ROIs, and the MTR_asym_ values were averaged over the generated ROIs. In addition, a 6‐pool Lorentzian multi‐peak model was fitted to averaged Z‐spectrum data per ROI. Between‐group (CI vs. CN) CEST effects were evaluated using Mann–Whitney *U* tests.

**Results:**

Motion correction reduced artifacts and visually improved CEST results. Multiple cortical and white matter ROIs showed differences in MTR_asym_ values between CI and CN groups (*p* < 0.05). Overall, the mean MTR_asym_ and Lorentzian amplitudes were lower in the CI than in the CN group.

**Conclusion:**

Our proposed pipeline enables robust data analysis using either MTR_asym_ or multi‐pool Lorentzian quantification approaches. The results suggest feasibility for detecting CEST differences between CI and CN groups in brain regions previously implicated in AD, motivating future validation in larger cohorts.

## 1 Introduction

Alzheimer's disease (AD) poses a significant public health challenge with an economic burden in the United States alone projected to exceed one trillion dollars by 2050 [1]. Currently, the clinical diagnosis of AD involves a combination of medical history assessment and cognitive testing, with support from a variety of diagnostic tools, including Magnetic Resonance Imaging (MRI), Positron Emission Tomography (PET), and cerebrospinal fluid (CSF) analysis [2]. Methods that biologically confirm the diagnosis of AD are essential in the era of targeted molecular therapies such as anti‐amyloid agents. Unfortunately, existing tests are invasive (e.g., CSF biomarkers) or are limited due to high costs and requirements for highly specialized facilities as with PET. These limitations are further compounded if one considers repeat monitoring to gauge response to therapy. MRI‐based modalities offer wider accessibility and are already incorporated into the recommended monitoring protocols for FDA‐approved anti‐amyloid treatments such as lecanemab and donanemab [3, 4]. This provides a prime opportunity to develop and implement novel MRI‐based AD markers.

Chemical exchange saturation transfer (CEST) MRI is a non‐invasive molecular imaging technique that shows promise in detecting molecular changes in the brain. By measuring the transfer of saturation between exchangeable protons in a molecule of interest (or side chain of a macromolecule) with the surrounding water pool, CEST enables detection of the said molecule by inference [5, 6]. This enables the indirect detection of molecules present in concentrations that are too low for direct observation by conventional MRI approaches. Specific frequencies within the CEST‐measured Z‐spectra are associated with different functional groups including hydroxyls (˜1 ppm), amines/guanidinium (˜2 ppm), and amides (˜3.5 ppm, also known as Amide Proton Transfer weighted [APTw] imaging, which the FDA recently approved to help distinguish primary gliomas from metastatic disease [7]). Thus, information about specific functional groups, and the molecules they are associated with, can be measured in the brain in vivo [8]. Furthermore, CEST MRI provides contrast that is influenced not only by the concentration but also the state of the target molecules. This has particular relevance to AD monitoring, where native proteins such as tau and amyloid undergo conformational changes, including folding and aggregation [9, 10, 11, 12, 13, 14]. We therefore expect that pathological molecular alterations in AD may be detectable using non‐invasive CEST MRI methods [12, 13, 15, 16, 17, 18].

The overarching goal of our work is to develop and validate a non‐invasive imaging method for staging as well as determining treatment response in AD patients using CEST MRI. Recently, our group showed that CEST effects can be used to distinguish between healthy tau monomers and pathologic aggregated tau fibrils in vitro [13]. Previous CEST studies in AD mice models have shown promise for differentiating between AD and control groups [16, 19, 20, 21, 22]. Limited human studies have also been able to differentiate cognitively impaired (CI) from cognitively normal (CN) individuals utilizing specific portions of the CEST Z‐spectrum, primarily APTw [23, 24, 25, 26, 27, 28]. Importantly, to the best of our knowledge, prior human studies have not focused on biologically confirmed AD.

Vitally, the incorporation of CEST processing with accepted neuroimaging analysis tools, such as Advanced Normalization Tools (ANTs) [29, 30, 31, 32] and FreeSurfer (MGH, Boston) is notably lacking. Such integration would be advantageous as it would allow the use of widely established post‐processing (e.g., ANTs) methods in conjunction with CEST for more precise and reproducible results. In pursuit of this, we developed a CEST processing pipeline combining CEST analysis and standard neuroimaging processing tools. Our secondary goal was to apply it to preliminarily test our hypothesis that CEST can detect pathologic differences between CI patients with underlying biomarker confirmed AD pathology and CN patients. The pipeline offers a standardized approach to region‐of‐interest (ROI) definitions, applied here to study AD patients. Our preliminary results highlight the technical improvements achieved, as well as the potential of CEST MRI to delineate disease‐related molecular changes in AD.

## 2 Methods

### 2.1 Subjects

Eight subjects (four CI subjects (three with mild cognitive impairment (MCI), one with moderate dementia) and four age‐matched CN controls) were recruited under an IRB‐approved protocol to establish processing pipeline feasibility.

Subjects were classified as CI as diagnosed by a subspecialty‐trained neurologist at UT Southwestern's Memory Clinic. The inclusion criteria for CI group included (1) a cognitive status of MCI or moderate dementia and (2) AD CSF biomarker levels consistent with a diagnosis of AD using the Athena Diagnostics ADmark Test [33]. This test assesses the levels of phosphorylated tau (p‐tau181P), total tau (t‐tau), and Aβ42 along with the presence of the Apolipoprotein E2, E3, and E4 alleles in the CSF. A negative result is defined as total tau to Aβ42 and p‐tau to Aβ42 ratio values below cutoffs of 0.023 and 0.28, respectively; or an Aβ42 value above the test's measuring range, and is consistent with amyloid PET. Moreover, all our CI participants met the test's thresholds of p‐tau > 68 pg/mL and Aβ42 to t‐tau ratio (ATI) < 0.8, consistent with underlying AD pathology. Note that for this test, a borderline result is p‐tau 54–68 pg/mL and ATI between 0.8‐1.2, and a negative result is p‐tau < 54 pg/mL and ATI > 1.2.

### 2.2 Imaging

All 8 subjects were scanned using a 3T MRI scanner (Philips Ingenia, NL). A 3D T_1_ weighted gradient echo (GRE) sequence was acquired prior to the CEST sequence for anatomic correlation. The sequence parameters were: FOV 24 × 24 × 17 cm^3^, resolution 1 × 1 × 1 mm^3^ (recon to 0.4 × 0.4 × 1 mm^3^), and TR/TE = 6.5/2.9 msec. For the CEST experiments, a multi‐slice CEST GRE multi‐point Dixon (mDixon) sequence, representing an expansion of the technique previously introduced in reference [34], was used with TR/TE/ΔTE = 5.4/1.65/1.1 msec, 3 echoes, TFE factor = 112. The CEST acquisition parameters were in‐plane resolution = 2 × 2 mm^2^, slice thickness = 5 mm, no gap, and 10 slices with 5 cm of craniocaudal coverage. The specific CEST parameters for this sequence were: 40 × 50 ms hyperbolic secant pulses (total RF saturation duration of 2 s), B_1rms_ = 1.2 μT, enabled by alternated parallel transmission [35], and 23 equally spaced points along the Z‐spectrum (±6 ppm) plus a reference image volume (−1560 ppm), for a total scan time of 12 min.

### 2.3 Image Processing Pipeline

Standard vendor‐provided multi‐point Dixon post‐processing was utilized to generate six different types of images for each frequency offset: (i) the source images (a total of three magnitude images, one for each TE), (ii) the water‐only image, (iii) the fat‐only image, (iv) the in‐phase (IP) image, (v) the out‐of‐phase (OP) image, and (vi) the B_0_ map. The water‐only images were used exclusively for downstream CEST analyses, ensuring robust fat suppression and minimizing lipid contamination of the Z‐spectrum.

**FIGURE 1:**
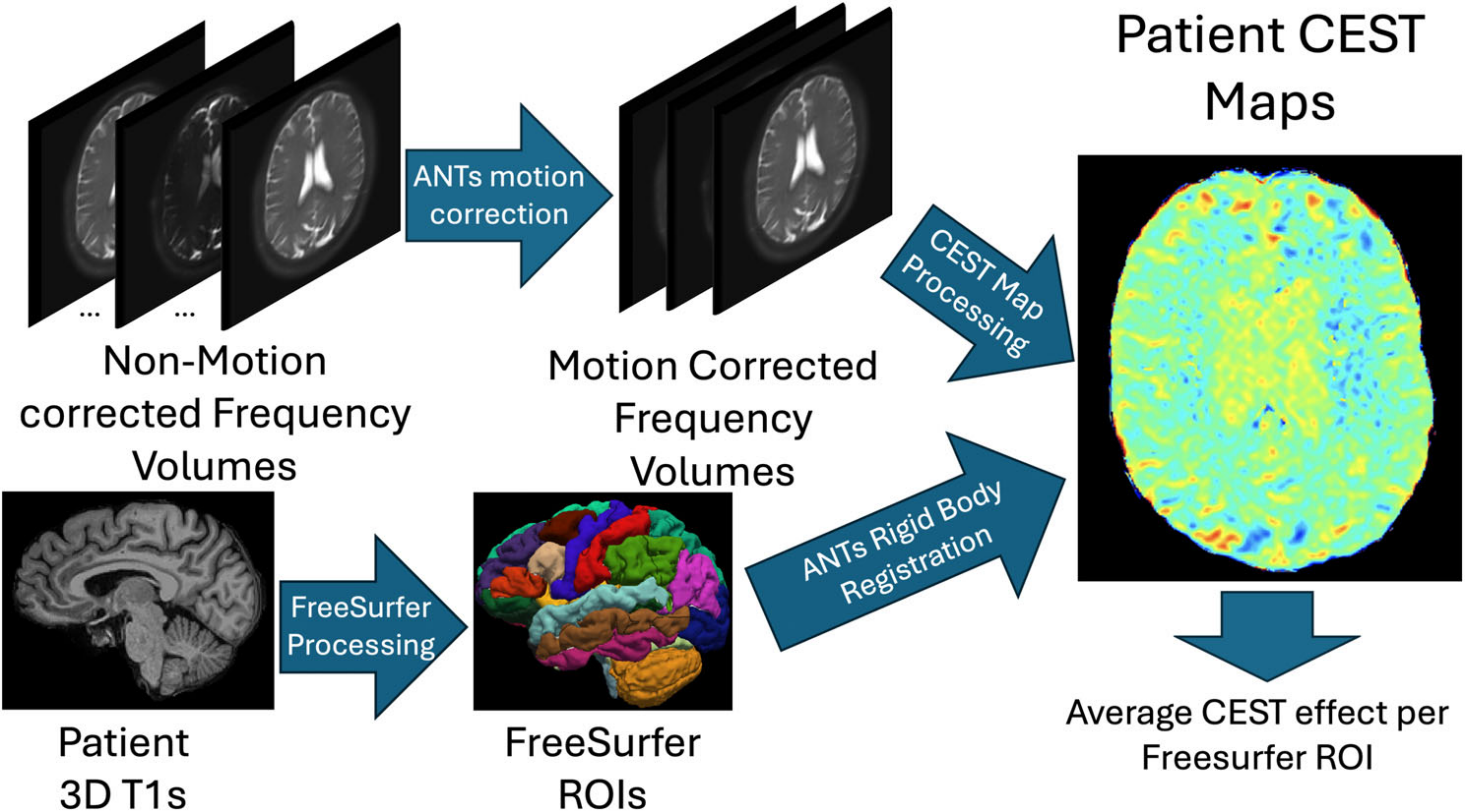
A schematic of the processing pipeline incorporating CEST and neuroimaging analysis tools. Water‐only frequency dependent images are motion corrected (ANTs) and used for MTR_asym_ map generation (at 1, 2, and 3.5 ppm). FreeSurfer 6.0 is used for processing and ROI generation, which are registered (ANTs Rigid Body) to CEST maps (MTR_asym_ or Lorentzian fit).

A visual summary of the processing pipeline is shown in Figure 1. First, for motion correction, the water‐only images were rigidly motion corrected with six degrees of freedom (3 translational and 3 rotational axes) using Advanced Normalization Tools (ANTs) [29]. The ANTs registration framework uses a mutual‐information cost function optimized via a symmetric diffeomorphic approach, which has been validated for inter‐modality MRI registration in elderly and neurodegenerative brain datasets [29, 30, 31, 32] This approach has been adopted in large‐scale neuroimaging projects, including ADNI and UK Biobank, demonstrating its robustness and suitability for the present study. As the Figure 1 schematic demonstrates, ANTs registration was used (i) to co‐register CEST frequency volumes to correct for motion; (ii) to register 3D T1w images to CEST images (more details below).

First, motion correction was accomplished by co‐registering all CEST frequency volumes to the reference volume using ANTs as described above. Note that since our CEST sequence achieved only 5 cm of craniocaudal coverage, these were partial brain volumes. Two zero padding slices were added to the top and bottom of each CEST frequency partial brain volume along the *z*‐axis allowing the partial brain volumes with patient motion in and out of plane to be reliably co‐registered to the reference volume. For brevity, we will continue referring to them as “volumes”.

After the abovementioned ANTs motion correction of the CEST volumes, custom MATLAB routines were used for a voxel‐wise Z‐spectrum interpolation, B_0_ correction using minimum of Z‐spectrum approach [36] and MTR_asym_ curve calculation. Three frequencies (1, 2, and 3.5 ppm) were used to generate MTR_asym_ maps for each subject on a voxel‐wise basis.

In parallel, the 3D T1w images were processed with Freesurfer 6.0 (MGH, Boston) to generate ROIs for each subject, and to ensure that there were comparable ROIs across all eight subjects [37]. ANTs rigid body registration was used to co‐register the subject specific ROIs to the subject specific CEST reference volume. To mitigate partial volume effects due to the lower in‐plane spatial resolution of CEST relative to the high‐resolution T1‐w anatomical images, we applied a rigid‐body co‐registration technique with six degrees of freedom (3 translational and 3 rotational axes), transforming each subject's T1‐w images to the native CEST space using a mutual information–based algorithm in ANTs as described above. This approach ensured that FreeSurfer‐derived ROIs were resampled directly into the lower‐resolution CEST space, preserving anatomical boundaries without artificially upsampling the CEST data. By aligning anatomical ROIs to the geometry of the CEST volumes, this strategy reduced the risk of partial volume contamination. The co‐registered images were also reviewed by two radiologists to ensure accuracy (AA and FY). This was done to ensure that residual motion or deformation did not lead to ROI misregistration or artifacts. None such cases were noted.

The co‐registered individual and composite FreeSurfer ROIs as well as CEST MTR_asym_ maps at 1, 2, and 3.5 ppm were then loaded into Python 3.8.18 using the Nibabel toolbox version 5.1.0 [37]. The FreeSurfer ROIs were applied to the MTR_asym_ maps to calculate an average MTR_asym_ value per FreeSurfer ROI per subject.

In addition to the MTR_asym_ analysis, a multi‐pool Lorentzian fit analysis was implemented, which better removes background MT and isolates different pool contributions [8]. This dual‐pathway design, incorporating both voxel‐wise MTR_asym_ maps and ROI‐based Lorentzian modeling, was intended to test pipeline adaptability to different quantification methods. Specifically, a 6 pool Lorentzian fit model, based on a previously described 5 pool Lorentzian fit model by Windschuh et al. [38], was fit to averaged Z‐spectral data over each FreeSurfer ROI. Given the limited SNR of single‐voxel Z‐spectra in this 3T pilot study, Lorentzian fitting was performed on ROI‐averaged spectra rather than voxel‐wise, as was previously demonstrated in multiple studies [39, 40, 41, 42]. This choice boosted the effective SNR (by √N across the ROI) and stabilized multi‐pool parameter estimates, mitigating non‐physiologic or highly variable fits that occurred in initial voxel‐level testing. The Lorentzian peak center positions were constrained to within ±0.01 ppm of literature‐reported values based on the 7T work by Windschuh et al. [38]. These tight priors were implemented to stabilize the fits in 3T data, where the lower spectral resolution and overlapping pool contributions otherwise made unconstrained fitting unreliable. While these constraints may limit flexibility for broader peaks such as MT or NOE, they were deemed appropriate here to achieve consistent fitting across subjects and to align the Lorentzian amplitudes with the traditional MTR_asym_ offsets. Future higher field or higher SNR studies may consider relaxing these priors to better characterize broad background pools. The original five pool model included water, semisolid MT, amine, amide pools and aliphatic rNOE pool [38]. The sixth pool corresponding to an approximate hydroxyl pool at 1 ppm was added so that comparisons could be made between the MTR_asym_ values at 1 ppm, 2 ppm, and 3.5 ppm. The initial, minimum, and maximum values for each Lorentzian parameter for amplitude, frequency position, and width can be found in Table S1. The fitting procedure broadly followed the steps described in reference [38]. Specifically, prior to ROI averaging, the Z‐spectra were interpolated with B_0_ correction on a voxel‐by‐voxel basis using the minimum of the Z‐spectrum (same step as used for the MTR_asym_ calculation).

### 2.4 Statistical Analysis

Given that the volumes acquired were partial brain volumes, there were regions that were not common to all subjects. Therefore, only the FreeSurfer ROIs that were common to all subjects were included in the analysis. The common ROIs that are bilateral in nature were averaged together and treated as single ROIs. Additionally, ROIs within the ventricular system were not included in this analysis as their signal was unreliable due to CSF pulsation throughout the acquisition [43].

A total of 75 ROIs were identified consistently across all 8 subjects which are listed in Table S2. These regions included bilateral ROIs known to be involved in AD progression that are routinely studied by researchers quantifying Aβ or tau burden with PET imaging [44, 45, 46]. White matter regions were also included in the analysis, since prior studies have indicated abnormal accumulation of pathologic proteins in these areas [47, 48, 49, 50].

Furthermore, as an additional evaluation and to align our analyses with commonly used regional groupings in the AD literature, we combined the FreeSurfer ROIs described above into established composite AD regions [51, 52]. Composite masks were generated for the medial temporal lobe (hippocampus, parahippocampal gyrus, and entorhinal cortex), lateral temporal lobe (middle, superior and inferior temporal gyri), lateral parietal lobe (superior and inferior parietal lobules), and medial parietal lobes (precuneus and posterior cingulate).

Due to the small pilot sample size which precluded testing for distributional normality, between‐group CEST effects (CI vs. CN) across each ROI for the MTR_asym_ maps and ROI‐based Lorentzian metrics were evaluated using the non‐parametric Mann–Whitney *U* test. Given the exploratory nature of this pilot study, no formal correction for multiple comparisons was applied. Accordingly, reported *p*‐values should be interpreted in the context of hypothesis generation rather than confirmation.

## 3 Results

### 3.1 Patient Demographics

The demographic information of the study participants can be found in Table 1. Three out of the four CI patients were diagnosed with MCI, and one was diagnosed with moderate dementia.

**TABLE 1:** Patient demographics.

Subject	Sex	Age	Group	Cognitive status
1	Male	58	CI	MCI
2	Male	60.5	CI	MCI
3	Male	61.8	CI	MCI
4	Male	62.5	CI	Moderate dementia
5	Female	56.2	CN	Normal
6	Male	60.9	CN	Normal
7	Female	64.6	CN	Normal
8	Male	75	CN	Normal

Abbreviations: CI, cognitively impaired; CN, cognitively normal; MCI, mild cognitive impairment.

### 3.2 Imaging Results

**FIGURE 2:**
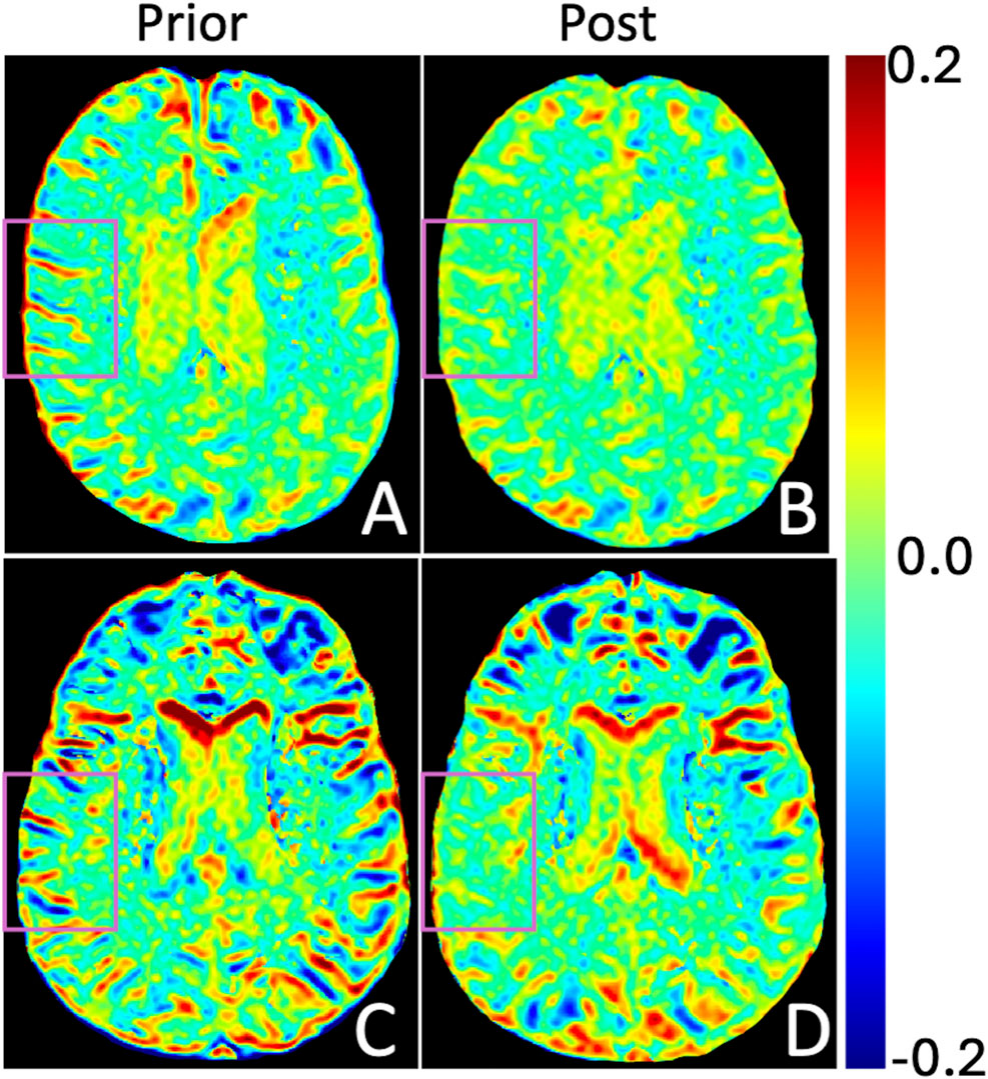
Single axial slice from an MTR_asym_ (3.5 ppm) map from representative cognitively normal (CN, A, B) and cognitively impaired (CI, C, D) participants: (A, C) prior to ANTs motion correction and (B, D) after ANTs motion correction, illustrating correction of erroneous motion‐related signals near gyral and sulcal boundaries. Light purple boxes outline brain regions with reduced artifacts following motion correction.

**FIGURE 3:**
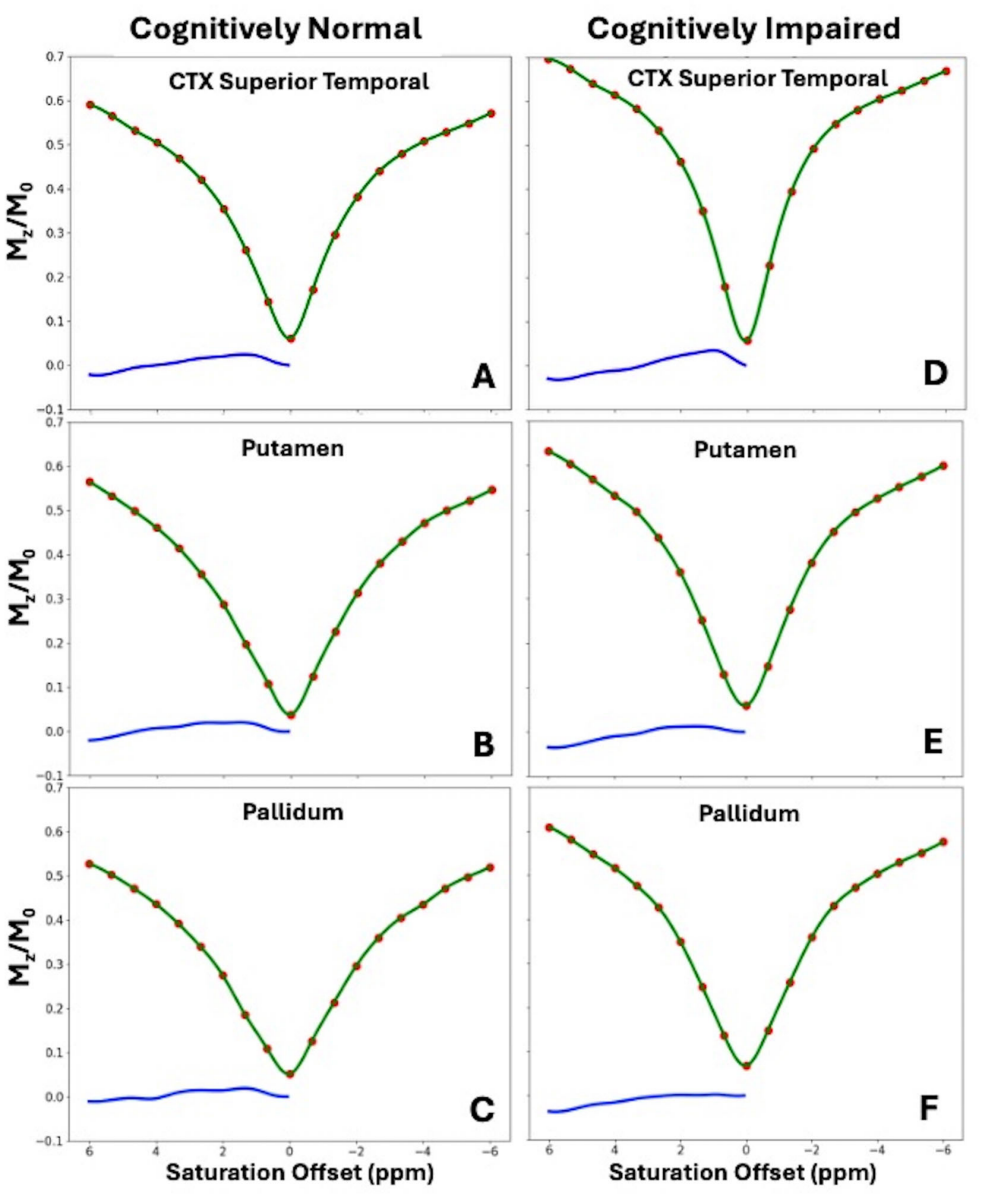
Representative ROI averaged MTR_asym_ curves from a Cognitive Normal (A–C) and a Cognitively Impaired (D–F) patients: (A, D) CTX Superior Temporal; (B, E) Putamen; (C, F) Pallidum. Red dots show raw data, green lines interpolated and B_0_ corrected Z‐spectra and blue line shows MTR_asym_.

The processing pipeline described in the methods section was used to process the acquired imaging data. Figure 2 displays ANTs motion‐corrected vs. non‐corrected MTR_asym_ (3.5 ppm) maps from a representative CN and CI subjects. It is evident that motion correction reduces erroneous signals, particularly along the gyral borders that would contaminate gray matter metrics. Representative MTR_asym_ curves from CI and CN patients from several ROIs are shown in Figures 3 and S1.

**FIGURE 4:**
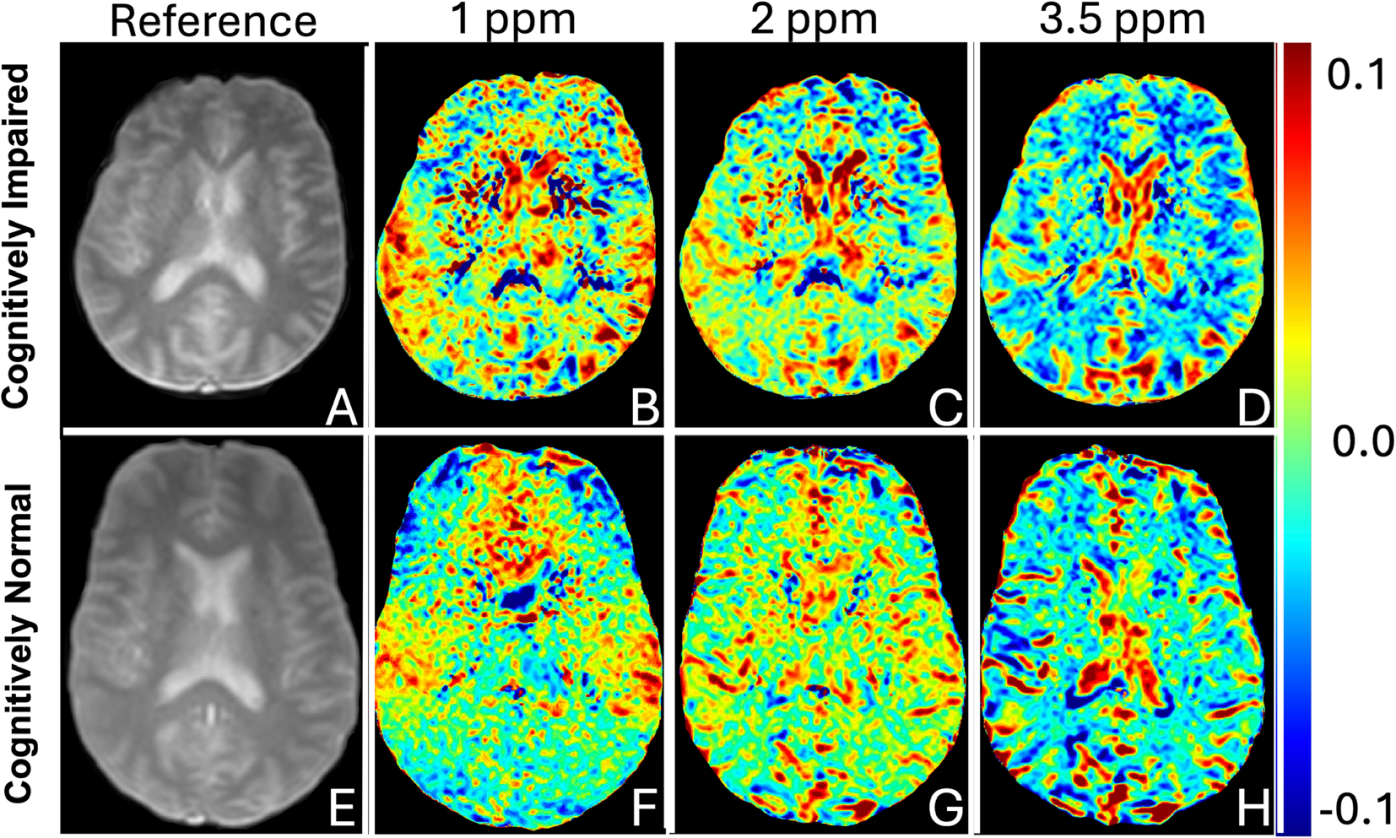
Representative examples from the cognitively impaired (CI) (A–D) and CN (E–H) groups showing a reference image for anatomy (A, E) and the calculated MTR_asym_ maps at 1 ppm (B, F), 2 ppm (C, G), and 3.5 ppm (D, H). All maps shown are post motion correction. Note narrower dynamic range of the scale (−0.1 to 0.1) compared to Figure 2 (−0.2 to 0.2).

MTR_asym_ maps were calculated for each CI patient and CN control on a voxel‐by‐voxel basis. Figure 4 displays representative results for CI and CN, showing reference images and MTR_asym_ maps at 1 ppm, 2 ppm, and 3.5 ppm. All the results shown are post motion correction. The same figure but without the motion correction processing step is provided in the Figure S2 to illustrate the improvement in cortical and subcortical alignment following the correction. Note the narrower display range (−0.1 to 0.1) used in this Figure compared with Figure 2 (displayed at −0.2 to 0.2), which was applied to enhance visualization of subtle regional CEST differences between CN and CI cases.

**FIGURE 5:**
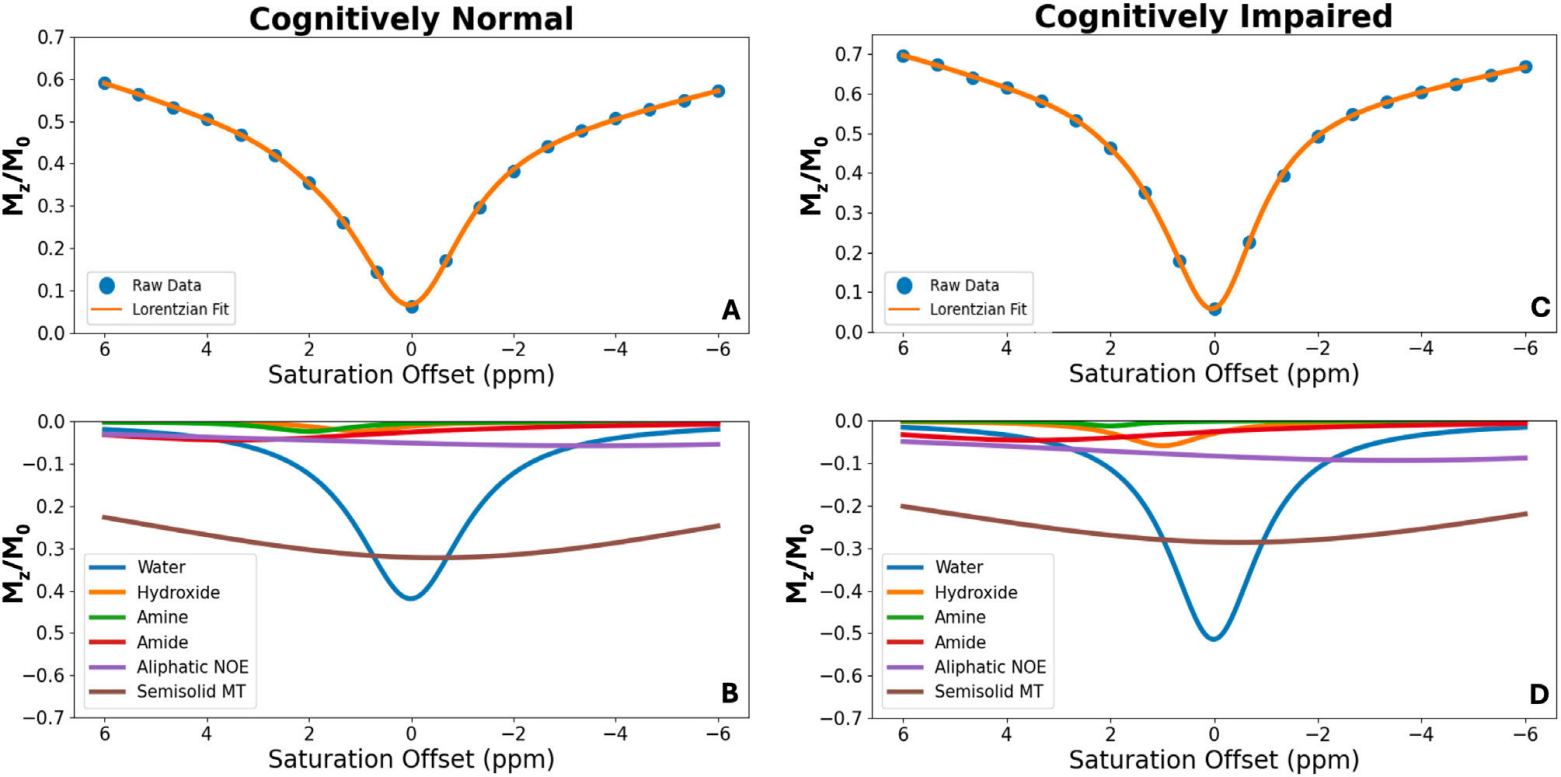
Representative results of the Lorentzian multi‐peak fitting of cognitively normal (CN) (A, B) and cognitively impaired (CI) (C, D) patients for the left hemisphere superior temporal cortex ROI. A and C show source Z‐spectra points (blue dots) and the resulting fitted curve (orange line). B and D demonstrate individual Lorentzian peaks that make up the multi‐Lorentzian curves in A and C: Water (blue), hydroxyl (orange), amine (green), amide (red), aliphatic NOE (lilac) and MT pool (brown). The coefficient of determination (*R* ^2^) was calculated as 1 minus the ratio of residual sum of squares to total sum of squares between the measured and fitted data: *R* ^2^ = 1.000. The root mean square error (RMSE) was calculated as the square root of the mean squared differences between the measured data and the Lorentzian fit: RMSE = 0.0031 and 0.0022 for A and C, respectively.

Representative multi‐peak Lorentzian curve ROI‐averaged fit analyses with individual peaks are shown in Figure 5.

### 3.3 Statistical Analysis Results

**FIGURE 6:**
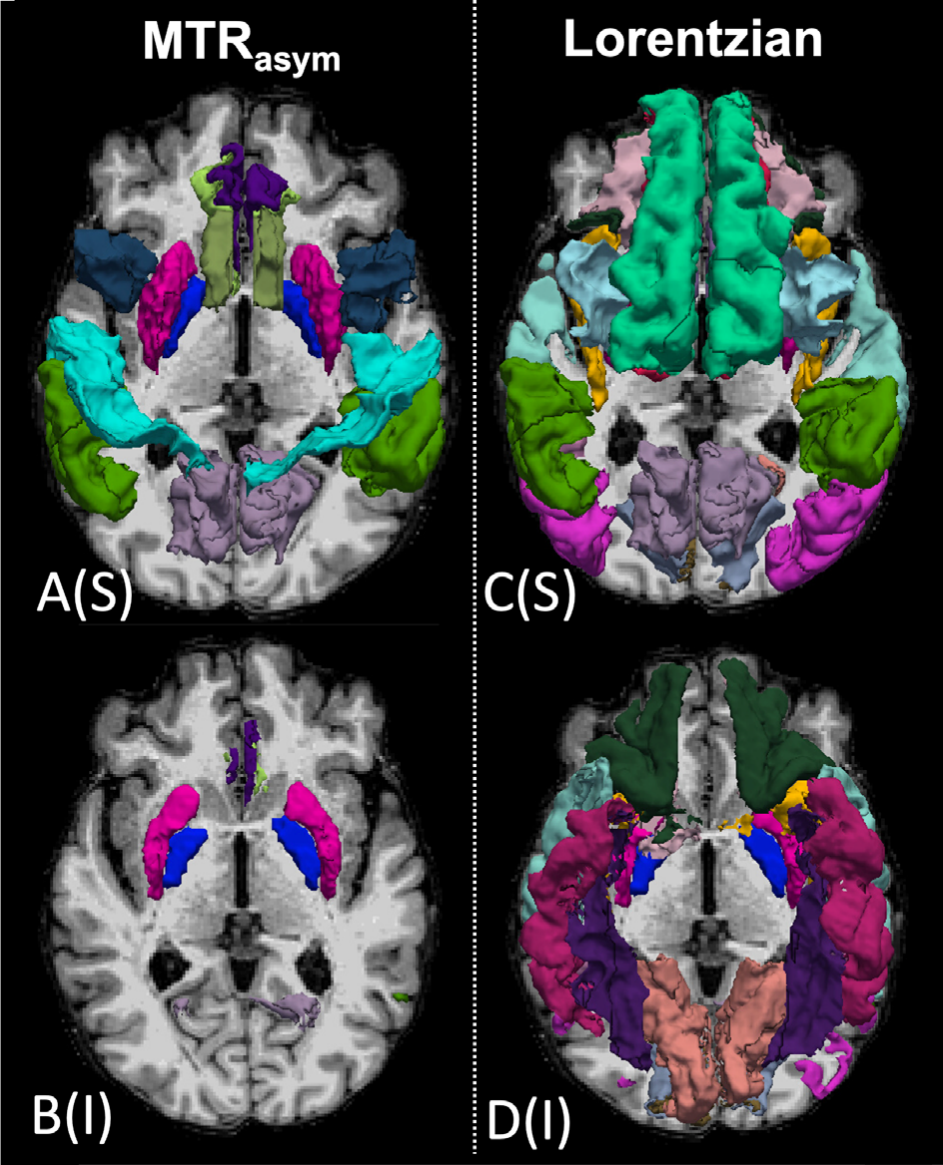
Axial T1‐weighted MRI slice overlaid with 3D representations of FreeSurfer ROIs identified as having uncorrected *p*‐values < 0.05 for group comparisons between cognitively impaired (CI) and cognitively normal (CN) participants. Panels A and B show differences from MTR_asym_ analysis; panels C and D show differences from Lorentzian peak analysis. Left and right subpanels display superior (S) and inferior (I) views, respectively.

Statistical comparisons were performed to illustrate the pipeline's ability to detect plausible group‐level trends, acknowledging that definitive conclusions are precluded by the pilot sample size. First, a group‐wise comparison was performed using the MTR_asym_ maps. The FreeSurfer ROIs with uncorrected *p*‐values less than 0.05 can be found in Table 2 and in Figure 6A,B. Multiple cortical and white matter ROIs showed differences between CI and CN groups (*p* < 0.05). In most of the ROIs, the mean MTR_asym_ in the CI group was lower than in the CN group at 2 and 3.5 ppm, and greater at 1 ppm.

**TABLE 2:** A table of FreeSurfer ROIs with uncorrected *p*‐values less than 0.05 between CI and CN groups using MTR_asym_ analysis.

MTR_asym_ map	FreeSurfer region	U‐statistic	*p*	CI mean	CN mean
3.5 ppm	Putamen	0	0.029	−0.021	−0.004
2 ppm	ctx‐rostral anterior cingulate	0	0.029	0.018	0.026
2 ppm	wm‐postcentral	0	0.029	0.003	0.019
3.5 ppm	Pallidum	0	0.029	−0.021	−0.008
3.5 ppm	wm‐rostral anterior cingulate	0	0.029	−0.025	−0.01
3.5 ppm	wm‐postcentral	0	0.029	−0.025	−0.007
3.5 ppm	wm‐parsopercularis	0	0.029	−0.029	−0.014
3.5 ppm	ctx‐precuneus	0	0.029	−0.008	0.001
2 ppm	Pallidum	0	0.029	0.002	0.015
2 ppm	Putamen	0	0.029	0.001	0.016
3.5 ppm	ctx‐supramarginal	0	0.029	−0.01	−0.003
2 ppm	wm‐caudalanteriorcingulate	0	0.029	0.006	0.03

Abbreviations: CI, cognitively impaired; CN, cognitively normal; CTX, cortex; WM, white matter.

Next, a group‐wise comparison was performed using the ROI‐averaged amplitudes of the Lorentzian peaks. FreeSurfer ROIs with *p*‐values less than 0.05 can be found in Table 3 and are also shown in Figure 6C,D. Similar to the MTR_asym_ results, the general trend was that the Lorentzian peak amplitudes were lower in the CI group compared to the CN group at 2 and 3 ppm, and higher at 1 ppm. Relative to the MTR_asym_ analysis, group‐level differences were notably more spatially widespread with the Lorentzian analysis. Again, all *p*‐values are uncorrected exploratory results due to the pilot sample size and should be interpreted accordingly.

**TABLE 3:** FreeSurfer ROIs with uncorrected *p*‐values < 0.05 for differences between cognitively impaired (CI) and cognitively normal (CN) groups using Lorentzian multi‐peak fitting analysis.

Lorentzian amplitude	FreeSurfer region	U‐statistic	*p*	CI mean	CN mean
2 ppm	ctx‐caudalanteriorcingulate	0	0.029	0.017	0.04
1 ppm	ctx‐inferiorparietal	16	0.029	0.036	0.009
2 ppm	ctx‐inferiortemporal	0	0.029	0.021	0.05
1 ppm	ctx‐insula	16	0.029	0.028	0.004
1 ppm	ctx‐lateralorbitofrontal	16	0.029	0.025	0.009
3.5 ppm	ctx‐lingual	0	0.029	0.018	0.055
3.5 ppm	ctx‐pericalcarine	0	0.029	0.008	0.039
1 ppm	ctx‐precuneus	16	0.029	0.016	0.001
2 ppm	ctx‐superiorfrontal	0	0.029	0.015	0.025
1 ppm	ctx‐superiortemporal	16	0.029	0.057	0.028
3.5 ppm	ctx‐supramarginal	0	0.029	0.014	0.022
2 ppm	Pallidum	0	0.029	0.021	0.04
−3.5 ppm	Pallidum	16	0.029	0.086	0.007
2 ppm	Putamen	0	0.029	0.02	0.033
−3.5 ppm	Putamen	16	0.029	0.09	0.018
3.5 ppm	wm‐bankssts	16	0.029	0.021	0.005
2 ppm	wm‐caudalmiddlefrontal	0	0.029	0.014	0.034
2 ppm	wm‐fusiform	0	0.029	0.016	0.031
1 ppm	wm‐lateralorbitofrontal	16	0.029	0.027	0.013
3.5 ppm	wm‐pericalcarine	0	0.029	0.014	0.025
2 ppm	wm‐superiorfrontal	0	0.029	0.015	0.026

Abbreviations: CTX, cortex; WM, white matter.

Our complementary analysis using the composite ROIs revealed differences in the hydroxyl (1 ppm) Lorentzian amplitude within the medial parietal lobe (Table S3), consistent with the findings from our more granular full ROI‐based analysis (Table 3).

## 4 Discussion

Herein, we describe a CEST processing pipeline that incorporates rigid motion correction and two different CEST analysis methods: (i) voxel‐by‐voxel MTR_asym_ calculation with subsequent MTR_asym_ maps at multiple frequency offsets and (ii) ROI‐based six peak Lorentzian fit model. Utilizing these methods, we observed differences between the CI and CN groups in multiple brain regions, with predominantly decreased CEST effect at 2 and 3.5 ppm and increased CEST effect at 1 ppm (Table 4).

**TABLE 4:** FreeSurfer ROIs with uncorrected *p*‐values < 0.05 for differences between cognitively impaired (CI) and cognitively normal (CN) groups identified by both MTR_asym_ and Lorentzian multi‐peak fitting analyses.

Common region	MTR_asym_ map	U‐statistic	*p*	CI mean	CN mean	Lorentzian pool	U‐statistic	*p*	CI mean	CN mean
ctx‐supramarginal	3.5 ppm	0	0.029	−0.010	−0.003	3.5 ppm	0	0.029	0.014	0.022
Pallidum	2 ppm	0	0.029	0.002	0.015	2 ppm	0	0.029	0.021	0.040
Putamen	2 ppm	0	0.029	0.001	0.016	2 ppm	0	0.029	0.020	0.033

Abbreviations: CTX, cortex; WM, white matter.

The application of standard neuroimaging motion correction using widely available software (ANTs) is noteworthy as it led to a reduction of erroneous signals along gyral boundaries. It is well known that motion can compromise the accuracy of CEST data, and this fact is underscored in Figure 2. Moreover, maintaining signal fidelity within cortical regions is of major importance for AD as these are key sites of pathological change.

MTR_asym_ analysis is the most widely reported CEST analysis method. While it has limitations [53, 54], it performs robustly and is straightforward to implement. The differences in CEST signal in specific brain areas within the CI group (Figure 4) were observable on visual inspection throughout the brain. For example, for 2 and 3.5 ppm, we observed clearly decreased CEST asymmetry signal (Figure 4, more blue) within the representative CI patient which is most visually apparent in the white matter (more prominent in frontal regions at 2 ppm, but diffuse at 3.5 ppm). Detailed ROI‐based analysis confirmed overall decreased CEST in these affected brain regions (Figure 6A,B and Table 2).

Lorentzian multi peak fitting is considered a more advanced analysis method than MTR_asym_ and has been shown to be more repeatable across scanners [55]. Importantly, it allows separate analysis of the positive and negative frequency ranges around water, leading to clearer resolution of the contributions MTR_asym_ analyses stemming from NOE (−3.4 ppm) and CEST (3.5 ppm). It also removes direct water saturation and broad background MT. In this study, ROI‐based averaging of the Z‐spectra was performed prior to fitting in order to improve SNR. Previous studies have performed similar Lorentzian fit analyses over tumor ROIs [56]. This approach identified multiple brain ROIs showing differences in CEST metrics between CI and CN groups (Table 3/Figure 6C,D). Notably, multiple frequency ranges, including NOE (−3.4 ppm), showed between‐group differences. We recognize that tightly constrained peak offsets in the Lorentzian model may oversimplify the broad peaks of the semisolid MT and rNOE pools, which could vary more in vivo. This was a tradeoff to ensure stable fits in lower SNR and Z‐spectral resolution 3 T pilot data and to allow one‐to‐one comparison with MTR_asym_ results. Future studies could investigate relaxing these constraints, particularly at higher field strengths.

While both analyses identified several cortical and white matter regions with differences between CI and CN groups, multi‐peak Lorentzian fitting identified a greater number of differentiated ROIs than MTR_asym_ analysis (Table 3/Figure 6C,D vs. Table 2/Figure 6A,B). In addition to 1 ppm, 2 ppm and 3.5 ppm, Lorentzian fitting identified differences at −3.5 ppm attributed to NOE. Table 4 lists common ROIs identified in both analyses (asymmetry and Lorentzian fit) at the same frequency value. Notably, these regions included areas known to be involved in AD pathology, such as the supramarginal gyrus, putamen, and the pallidum [57, 58, 59, 60]. PET imaging studies have highlighted the orbitofrontal regions as being among the earliest areas to show increased Aβ radiotracer uptake during AD progression [61]. The ability to detect differences in these early‐affected brain regions suggests that CEST MRI holds promise as a biomarker for evaluating AD progression. Three of the four CI patients in this study met clinical criteria for MCI due to AD. Therefore, while these results are preliminary, it is encouraging that we observed differences in CEST measures in relatively early‐stage disease patients.

The additional analysis using aggregated ROIs reduced the number of statistical comparisons. But it also appeared to attenuate subtle local effects, possibly due to spatial averaging across heterogeneous subregions. This suggests that in early disease stages such as MCI, finer anatomical resolution may be advantageous for detecting more localized molecular alterations. Accordingly, our proposed pipeline preserves the flexibility to operate at both detailed ROI and aggregated‐region levels, allowing future studies with larger cohorts to balance statistical power with anatomical specificity.

A speculative area of discussion relates to the histopathology of AD. As noted, the distribution of significant ROIs generally aligned with the regional deposition of Aβ that has been observed posthumously in AD patients [50, 62], including the inferior frontal lobes, inferior temporal lobes, and inferior occipital lobes [62] as shown in Figure 6. In contrast, Braak et al. reported that hippocampal deposition of Aβ does not occur until the later stages of AD [62]. Given that our study primarily consisted of patients at earlier stages of AD (i.e., MCI), it follows that the reason we did not observe any group‐level differences in the hippocampi could be because they had not progressed to later stage AD.

The observed decrease in MTR_asym_ (3.5 ppm) could be associated with the protein misfolding, as was indicated in previous studies [12, 13, 14]. The increase at 1 ppm could also be associated with conformational changes, increased glycoprotein, neuroinflammation as well as “contamination” from increased water signal due to atrophy and tissue loss. To try to mitigate this possible confound, we incorporated several strategies into our analysis pipeline. First, high‐resolution 3D T1‐w anatomical images were coregistered to the lower‐resolution CEST data using mutual information–based rigid‐body registration in ANTs, allowing precise propagation of FreeSurfer‐defined ROIs into CEST space while preserving anatomical boundaries. Second, motion correction was applied with visual quality checks to ensure minimal residual misalignment. Third, we used water‐only images to limit contamination from lipid signals. Finally, the use of ROI‐level multi‐pool Lorentzian fitting should help to isolate molecular CEST contributions versus broad background macromolecular transfer and potential CSF contamination. Interestingly, in our recent in vitro study [13], we observed a similar trend of decreased MTR_asym_(3.5 ppm) and MTR_asym_ (2 ppm) as well as an increase of MTR_asym_(1 ppm) in diseased versus healthy tau protein. Increased NOE in the CI group could be associated with myelin disintegration and aliphatic molecules becoming transiently more mobile and enhancing the rNOE effect. Additional studies that would include animal models and pathology assessment are needed to more precisely decipher the exact origins of the observed changes.

Overall, our results demonstrate decreased MTR_asym_ (3.5 ppm and 2 ppm) in CI participants. The current literature is still limited and somewhat contradictory. Few previous human studies have reported elevated APTw signals in MCI and AD [23, 24, 25, 28] often attributed to increased mobile protein accumulation such as Aβ and tau aggregates. At the same time, more recent investigations, particularly in animal models of early‐stage AD, have observed reduced 3.5 ppm CEST signals [17, 22] (different metrics were used in different studies). Moreover, the recent review by Orzylowska and Oakden [18] concludes that: “Magnetization transfer ratio (MTR) … is consistently lower in AD subjects relative to healthy controls, particularly in the temporal lobe”. This conclusion is consistent with our study. Lastly, differences in acquisition parameters such as RF saturation power, duration, and choice of CEST metric may further contribute to variability across study results. These factors underscore the importance of considering disease stage, molecular state, and technical protocol differences when interpreting CEST measurements in neurodegenerative disease.

While this pipeline is compatible with both MTR_asym_ and multi‐pool Lorentzian quantification approaches, the choice of metric should depend on study goals and acquisition parameters. MTR_asym_ mapping may be preferable in clinically constrained protocols with limited frequency sampling and time constraints, given its robustness and computational simplicity. In contrast, Lorentzian multi‐pool fitting can offer superior specificity by separating overlapping pools and accounting for background magnetization transfer effects, albeit with increased modeling complexity and the need for higher SNR. Our pipeline can accommodate either one, enabling future investigators to select the approach most appropriate for future applications. It can also be integrated with other metrics, such as AREX [63].

It is important to acknowledge the limitations of our study. First, the small sample size limits the generalizability of the findings. Accordingly, these results should be interpreted as hypothesis‐generating rather than confirmatory. Additionally, because 75 ROIs were analyzed, the risk of type I error due to multiple comparisons is notable. We acknowledge that with the multiple tests, even uncorrected *p*‐values below 0.05 must be interpreted very cautiously. Formal correction methods such as Bonferroni were not feasible due to the limited sample size and coarse *p*‐value granularity of the non‐parametric tests. Therefore, we also did analysis of the aggregate ROIs, which overall confirm our results from more granular ROIs. While the differences observed here between CI and CN groups are highly encouraging, a larger cohort study is needed to ensure the reproducibility and accuracy of the results. Second, the brain coverage was incomplete and required additional processing steps. A different acquisition protocol, e.g., utilizing accelerated techniques and allowing full brain coverage [64], would enhance robustness and is currently being explored by our group.

Although we attempted to minimize partial volume effects through precise T1‐to‐CEST coregistration and propagation of anatomically defined ROIs into the native CEST space, along with manual inspection, the difference in spatial resolution remains a source of potential confounding. While our cohort primarily consisted of individuals in the early stages of Alzheimer's disease—who are less likely to exhibit pronounced brain atrophy—subtle cortical thinning or CSF expansion may still have influenced CEST metrics. We expect this issue may be even more pronounced in later‐stage AD patients with advanced atrophy. We acknowledge this limitation and note that future studies with higher‐resolution CEST acquisitions could further reduce these effects.

## 5 Conclusions

This work establishes a CEST processing pipeline incorporating standardized neuroimaging tools and applied it in a pilot study of cognitively impaired patients with underlying AD pathology. The results indicate CEST differences between CI and CN groups in multiple brain regions, aligning with known topography of AD pathology. The detection of changes in specific brain regions associated with AD supports the notion that CEST may offer a non‐invasive AD biomarker that can be readily implemented in standard‐of‐care baseline and safety monitoring AD MRI protocols. Our proposed pipeline allows for more robust data analysis, enhancing the potential for clinical translation of CEST MRI. Future work with larger cohorts and optimized acquisition protocols will be essential to validate these preliminary findings.

## Funding

This work was supported by Texas Alzheimer's Research & Care Consortium (1029408), NIH (R01CA252281, T32‐EB028093), and the Southwestern Medical Foundation and G.R.White Trust.

## Conflicts of Interest

Dr. Ivan Dimitrov and Dr. Jochen Keupp are employed by Philips.

## Supporting information


**Table S1:** Starting, lower, and upper bounds for each pool in Lorentzian fit analysis: chemical shift in ppm (Δ), Lorentzian amplitude (A) and Lorentzian width (Γ). The pools are identified by expected approximate ppm value and generic exchanging group.
**Table S2:** FreeSurfer regions‐of‐interest (ROIs) identified across all 8 subjects and included in the subsequent CEST analysis. CC—corpus callosum, CTX—cortex, WM—white matter.
**Table S3:** Aggregate ROIs with uncorrected *p*‐values < 0.05 for differences between cognitively impaired (CI) and cognitively normal (CN) groups using Lorentzian multi‐peak fitting analysis. Abbreviations: MPL = medial parietal lobes (comprised of the precuneus and posterior cingulate).
**Figure S1:** Additional examples of ROI averaged MTR_asym_ curves from a Cognitive Normal (A, B) and a Cognitively Impaired (C, D) patients: (A, C) CTX Rostral Anterior Cingulate; (B, D) CTX Precuneus. Red dots show raw data, green lines interpolated and B_0_ corrected Z‐spectra and blue line shows MTR_asym_. Abbreviations: CTX = cortex.
**Figure S2:** The analog of Figure 4, but without the motion correction step. Representative examples from the cognitively impaired (CI) (A–D) and CN (E–H) groups showing a reference image for anatomy (A, E) and the calculated MTR_asym_ maps at 1 ppm (B, F), 2 ppm (C, G), and 3.5 ppm (D, H). All maps shown are without motion correction.
