# Supplementary material for: CEST MRI Processing Pipeline in Pilot Study of Alzheimer's Disease Patients
Source: Magn Reson Med. 2026 Jan 4;95(5):2828–39. doi: 10.1002/mrm.70240 (PMC12962198; doi:10.1002/mrm.70240)
Supplement: Supplementary file 1 — Table S1: Starting, lower, and upper bounds for each pool in Lorentzian fit analysis: chemical shift in ppm (Δ), Lorentzian amplitude (A) and Lorentzian width (Γ). The pools are identified by expected approximate ppm value and generic exchanging group. Table S2: FreeSurfer regions‐of‐interest (ROIs) identified across all 8 subjects and included in the subsequent CEST analysis. CC—corpus callosum, CTX—cortex, WM—white matter. Table S3: Aggregate ROIs with uncorrected p‐values < 0.05 for differences between cognitively impaired (CI) and cognitively normal (CN) groups using Lorentzian multi‐peak fitting analysis. Abbreviations: MPL = medial parietal lobes (comprised of the precuneus and posterior cingulate). Figure S1: Additional examples of ROI averaged MTRasym curves from a Cognitive Normal (A, B) and a Cognitively Impaired (C, D) patients: (A, C) CTX Rostral Anterior Cingulate; (B, D) CTX Precuneus. Red dots show raw data, green lines interpolated and B0 corrected Z‐spectra and blue line shows MTRasym. Abbreviations: CTX = cortex. Figure S2: The analog of Figure 4, but without the motion correction step. Representative examples from the cognitively impaired (CI) (A–D) and CN (E–H) groups showing a reference image for anatomy (A, E) and the calculated MTRasym maps at 1 ppm (B, F), 2 ppm (C, G), and 3.5 ppm (D, H). All maps shown are without motion correction. [file MRM-95-2828-s001.docx]

**Supplementary Material**

| **Pool** | **Start Values (Δ, A, Γ)** | **Lower Boundaries (Δ, A, Γ)** | **Upper Boundaries (Δ, A, Γ)** |
| --- | --- | --- | --- |
| Water (0 ppm) | 0, -0.9, 1.4 | -0.01, -1, 0.3 | 0.01, -0.02, 10 |
| Hydroxide (1 ppm) | 1, -0.01, 1 | 0.99, -0.7, 0.4 | 1.01, 0, 1 |
| Amine (2 ppm) | 2, -0.01, 1 | 1.99, -0.5, 1 | 2.01, 0, 3.5 |
| Amide (3.5 ppm) | 3.5, -0.025, 0.5 | 3.49, -0.2, 0.4 | 3.51, 0, 4 |
| Aliphatic rNOE (-3.5 ppm) | -3.5, -0.02, 3 | -3.51, -0.6, 0.5 | -3.49, 0, 10 |
| Semisolid MT (-0.5 ppm) | -0.5, -0.1, 25 | -0.51, -1, 10 | -0.49, 0, 99 |

***Table S1.*** *Starting, lower, and upper bounds for each pool in Lorentzian fit analysis: chemical shift in ppm (***Δ***), Lorentzian amplitude (A) and Lorentzian width (***Γ***). The pools are identified by expected approximate ppm value and generic exchanging group.*

| **Freesurfer ROIs** | **Freesurfer ROIs** | **Freesurfer ROIs** |
| --- | --- | --- |
| Accumbens Area | ctx lingual | wm inferiorparietal |
| Amygdala | ctx medialorbitofrontal | wm inferiortemporal |
| Brain Stem | ctx middletemporal | wm insula |
| CC Anterior | ctx parahippocampal | wm isthmuscingulate |
| CC Central | ctx parsopercularis | wm lateraloccipital |
| CC Mid Anterior | ctx parsorbitalis | wm lateralorbitofrontal |
| CC Mid Posterior | ctx parstriangularis | wm lingual |
| CC Posterior | ctx pericalcarine | wm medialorbitofrontal |
| Caudate | ctx postcentral | wm middletemporal |
| Cerebellum Cortex | ctx posteriorcingulate | wm parahippocampal |
| Hippocampus | ctx precentral | wm parsopercularis |
| Pallidum | ctx precuneus | wm parsorbitalis |
| Putamen | ctx rostralanteriorcingulate | wm parstriangularis |
| Thalamus Proper | ctx rostralmiddlefrontal | wm pericalcarine |
| VentralDC | ctx superiorfrontal | wm postcentral |
| ctx bankssts | ctx superiorparietal | wm posteriorcingulate |
| ctx caudalanteriorcingulate | ctx superiortemporal | wm precentral |
| ctx cuneus | ctx supramarginal | wm precuneus |
| ctx frontalpole | ctx transversetemporal | wm rostralanteriorcingulate |
| ctx inferiorparietal | wm bankssts | wm rostralmiddlefrontal |
| ctx inferiortemporal | wm caudalanteriorcingulate | wm superiorfrontal |
| ctx insula | wm caudalmiddlefrontal | wm superiorparietal |
| ctx isthmuscingulate | wm cuneus | wm superiortemporal |
| ctx lateraloccipital | wm frontalpole | wm supramarginal |
| ctx lateralorbitofrontal | wm fusiform | wm transversetemporal |

***Table S2.*** *FreeSurfer regions-of-interest (ROIs) identified across all 8 subjects and included in the subsequent CEST analysis.* CC – corpus callosum, CTX – cortex, WM – white matter.

| **Lorentzian Amplitude** | **Aggregate Region** | **U-Statistic** | **P-value** | **CI mean** | **CN mean** |
| --- | --- | --- | --- | --- | --- |
| 1 ppm | MPL | 16 | 0.029 | 0.019 | 0.002 |

***Table S3.*** *Aggregate ROIs with uncorrected p-values <0.05 for differences between cognitively impaired (CI) and cognitively normal (CN) groups using Lorentzian multi-peak fitting analysis.* ***Abbreviations: MPL = medial parietal lobes (comprised of the precuneus and posterior cingulate).***

**
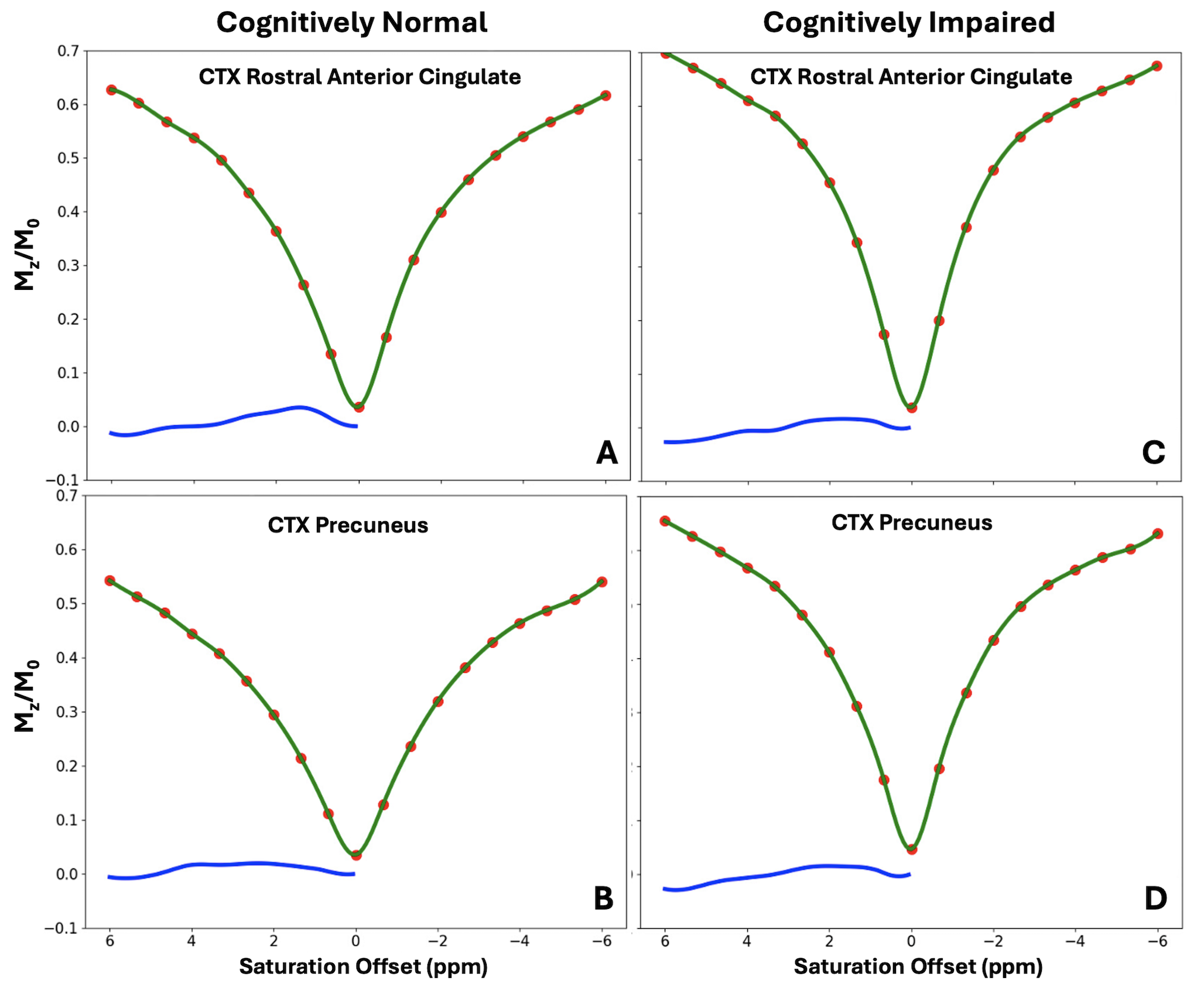
**

***Figure S1.*** *Additional examples of ROI averaged MTR_asym­_ curves from a Cognitive Normal (A,B) and a Cognitively Impaired (C,D) patients: (A,C) CTX Rostral Anterior Cingulate; (B,D) CTX Precuneus. Red dots show raw data, green lines interpolated and B_0_ corrected Z-spectra and blue line shows MTR_asym_. Abbreviations: CTX = cortex.*

***
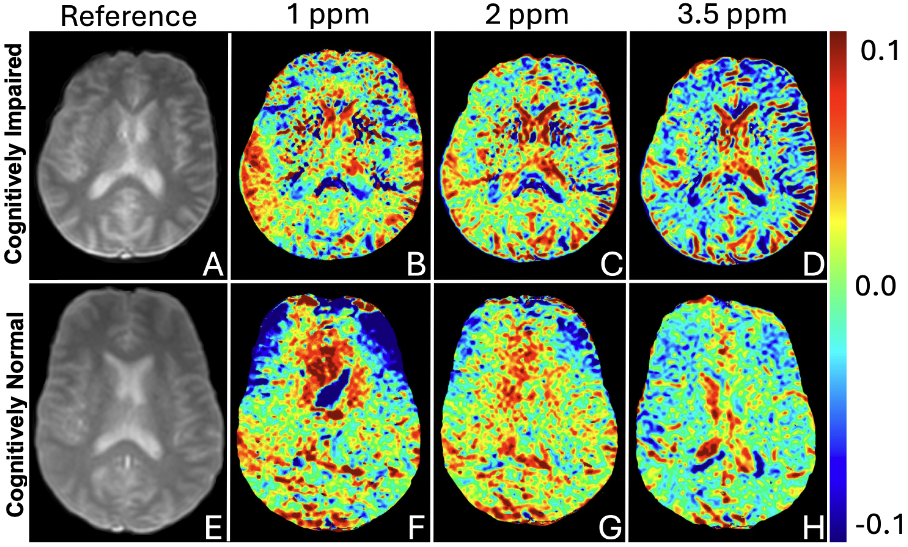
Figure S2.*** *The analogue of figure 4, but without the motion correction step. Representative examples from the cognitively impaired (CI) (A-D) and CN (E-H) groups showing a reference image for anatomy (A,E) and the calculated MTR_asym_ maps at 1 ppm (B,F), 2 ppm (C,G), and 3.5 ppm (D,H). All maps shown are without motion correction.*
